# Malignant breast adenomyoepithelioma: case report and literature review

**DOI:** 10.3389/fmed.2024.1525050

**Published:** 2025-01-15

**Authors:** Jinxiu Ma, Gang Zhao, Mingchao Bi, Xiaoxiao Dong, Jian Sun, Xiaozhen Wang

**Affiliations:** ^1^Department of Breast Surgery, General Surgery Center, The First Hospital of Jilin University, Changchun, China; ^2^Department of Ophthalmology, The First Hospital of Jilin University, Changchun, China

**Keywords:** adenomyoepithelioma, breast cancer, diagnosis, treatment strategies, pathology

## Abstract

Malignant adenomyoepithelioma (MAME) of the breast is a rare tumor with both benign and malignant features. We report a case of a 67-year-old woman who presented with a mass in the outer quadrant of the right breast, detected during a routine check-up. The mass was classified as BI-RADS 3. After minimally invasive excision, pathology confirmed low-grade malignant adenomyoepithelioma. The patient then underwent total mastectomy and sentinel lymph node biopsy (SLNB). At two years of follow-up, there was no recurrence or metastasis. This case highlights the rarity of MAME and the importance of early diagnosis and complete excision.

## Introduction

Breast adenomyoepithelioma (AME) was clearly defined by the WHO in 2019 as a lesion consisting of distinct, solidly proliferating myoepithelial cells. It is an extremely unusual type of breast epithelial tumor. AME is characterized by bi-directional differentiation of mammary epithelial cells and myoepithelial cells (1), and exhibits a diverse range of biological behaviors, including recurrence, metastatic potential, and malignant transformation. According to their clinicopathological features and biological behavior, AME can be classified as benign, atypical and malignant (*in situ* and invasive). Malignant adenomyoepithelioma (MAME), also referred to as AME with carcinoma, arises from malignant transformation in one of its cellular components, typically the myoepithelial cells. MAME is a particularly rare entity, accounting for only 1% of primary breast carcinomas (2). Given its rarity and the potential for misdiagnosis, this report presents a case of malignant AME, emphasizing its distinctive clinical and pathological features. The case contributes to the limited knowledge of MAME, providing insights into its presentation, diagnostic challenges, and management implications. The study aims to improve recognition and understanding of MAME to guide clinical practice and enhance patient outcomes.

## Case report

**Figure 1:**
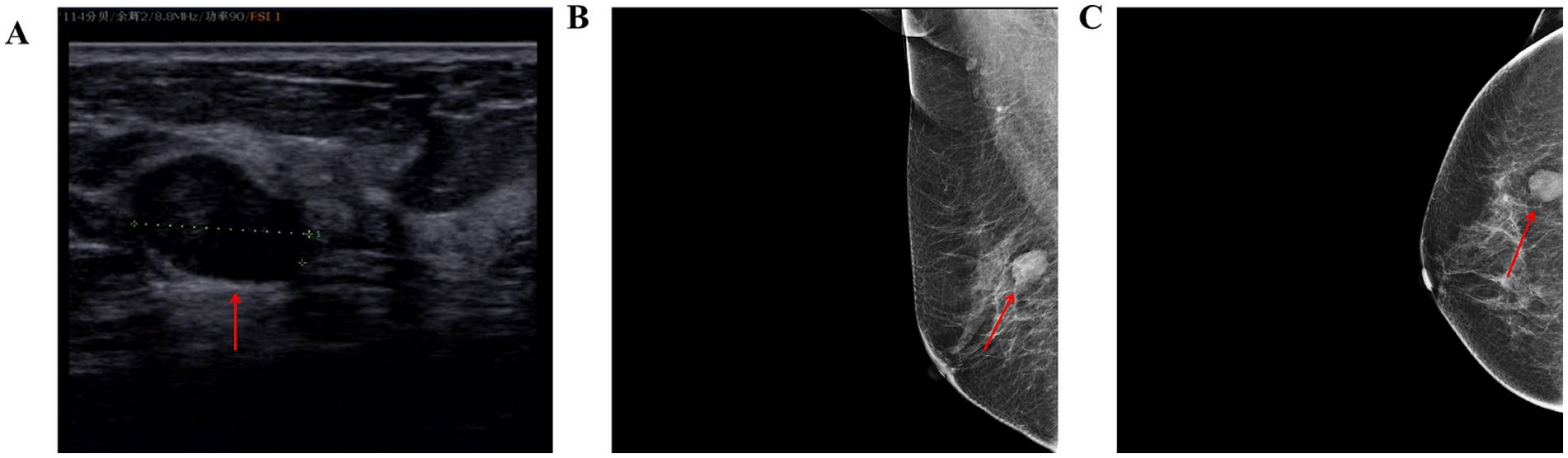
**(A)** Color Doppler ultrasound of the breast shows a 17.4 × 12.9 mm mass at the 9 o’clock position of the right breast, with a clear border and irregular shape. **(B,C)** Mammograms of the patient (red arrows representative lesion).

The patient is a 67-year-old female. During a routine medical check-up at our hospital, a Doppler ultrasound revealed a 17.4 × 12.9 mm mass in the outer quadrant of the right breast (Figure 1A), which was classified as BI-RADS category 3. Mammography (Figures 1B,C) showed a 2.0 cm × 1.3 cm well-defined mass with clear margins in the right breast, also classified as BI-RADS category 3. Shadows of lymph nodes were observed bilaterally in the axillary regions, with slightly increased density. The patient had no history of surgery and trauma, and had a normal menstrual history. There was no remarkable personal or family history. In the outpatient clinic, she was diagnosed with a right breast mass. Given the malignant potential of approximately 2% for Bi-rads 3 graded tumors, the patient strongly requested surgical excision. Subsequently, she underwent a local anesthesia-assisted minimally invasive rotational excision of the right breast mass at our hospital.

**Figure 2:**
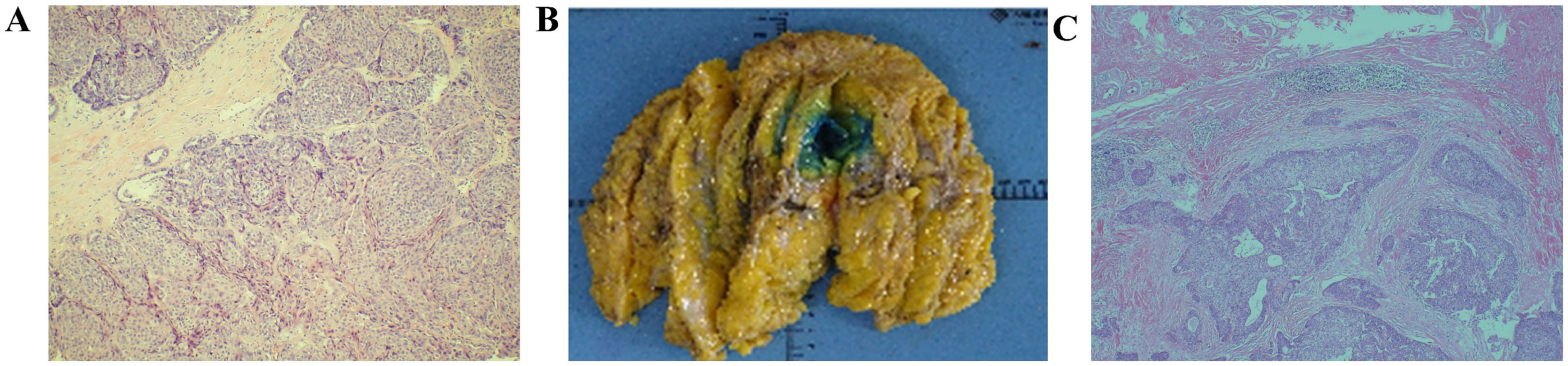
**(A)** Tumor nests with mildly heterogeneous cells; nucleoli 1–8 per HPF (HE ×100) **(B)** General view of postoperative specimens. **(C)** Fusiform and epithelioid hyaline myoepithelial cells, and cuboidal or columnar glandular epithelial cells forming tubular or papillary structures (HE × 40).

Postoperative paraffin pathology (Figure 2A) revealed low-grade malignant adenomyoepithelioma, characterized by a nested mass with peripheral pushing and infiltrative growth. Mild cellular atypia was noted, with a nuclear grade of 1–8/10 HPF. The excised tissue was fragmented, and the tumor size should be evaluated using additional imaging studies. Immunohistochemical analysis showed: Ki-67 (+10%), ER (−), PR (−), Her-2 (0), AR (−), S100 (+), CK5/6 (+), p63 (+), CK8/18 (+), Calponin (−), Syn (−), CgA (−), E-Cadherin (+).

The patient was readmitted for further management. On physical examination, a 0.5 cm surgical scar was noted at the 9 o’clock position on the right breast, which was healing well. No obvious enlargement of either breast was observed, and no significant lymphadenopathy was detected in the axillae or supraclavicular regions bilaterally. Routine preoperative examinations revealed no abnormalities.

A total mastectomy with sentinel lymph node biopsy (SLNB) of the right breast was successfully performed. Postoperative paraffin pathology (Figures 2B,C) revealed a small residual area of low-grade malignant adenomyoepithelioma in the excised breast specimen, identified through serial sections and immunohistochemistry. The maximum diameter of the residual tumor was approximately 0.1 cm, in line with previous pathological findings. The defect was located in the outer lower quadrant of the breast, measuring approximately 1.9 cm × 1.8 cm × 1.5 cm, surrounded by fibrous tissue hyperplasia with areas of hemorrhage. There was no tumor infiltration into the vasculature or nerves, and no tumor was found in the nipple, superficial fascia, or remaining quadrants of the breast tissue. Intraoperative evaluation of the right sentinel lymph node revealed no evidence of metastasis (0/1). Immunohistochemical examination showed a low proliferative index (Ki-67 + 1%), with positive markers for CK5/6, S-100, and p63 in the residual tumor area, supporting the diagnosis of low-grade malignant adenomyoepithelioma.

After the surgery, the patient did not undergo any other adjuvant treatment and was regularly reviewed. She returned for a follow-up examination at our hospital, where all results, including breast ultrasound, mammography of the left breast, abdominal ultrasound, lymph node ultrasound, lung CT, and tumor markers, were negative. No signs of recurrence or metastasis were found (Table 1).

**Table 1:** Patient treatment timeline.

Date	Event	Description
08/12/2021	Physical examination and imaging	Palpable breast mass, imaging showed atypical features, sugery recommended
08/20/2021	Mammotome Operation	Diagnosed as MAME
09/03/2021	Surgical treatment	Total mastectomy and axillary lymph node dissection
09/15/2022	Follow-up examination	Routine follow-up, CT and breast ultrasound showed no recurrence
07/01/2023	Continued follow-up	Annual check-up, stable condition, no tumor recurrence

## Discussion

Adenomyoepithelioma (AME) is an exceedingly rare group of tumors, first described by Bhatkule et al. (3) and Pradhan and Yadav (4). It is thought to arise from stem cells with intermediate epithelial/myoepithelial differentiation within the terminal ductal lobular units (TDLU) of the breast, though the precise histogenesis and etiology remain unclear. Tumor development generally progresses from adenopathy with or without myoepithelial hyperplasia, to benign AME, and in some cases, to malignant forms such as pure myoepithelial carcinoma or AME with malignant components (1, 4, 5). In long-standing adenopathies, fibroadenomas, or other benign lesions, AME may also result from myoepithelial overgrowth (6).

MAME can occur in individuals ranging from 22 to 92 years old, with a median age of onset around 58 years. It is more common in postmenopausal women and less frequently reported in men. The majority of cases are sporadic, with no familial clustering (6, 7). In the present case, the patient was a 67-year-old female with no significant family medical history. Primary MAME tumors typically develop in the lateral breast, although a few cases have been reported beneath the areola, which may present with nipple discharge. The tumors are usually well-defined, regular in shape, and characterized by slow, painless growth, though they can occasionally exhibit rapid expansion. Because of the rarity of these tumors, there is limited literature describing the radiologic appearances. Imaging findings are often non-specific, with the tumor appearing as a round, lobulated, cystic solid, or dense mass, often resembling a breast fibroma, which can lead to diagnostic confusion.

Histologically, MAME is classified into three main types: malignant AME *in situ*, invasive malignant AME, and AME associated with invasive carcinoma. Diagnosis of invasive MAME requires the following criteria: (1) hyperproliferation of adenoepithelial or myoepithelial components; (2) significant cellular atypia; (3) infiltrative growth; (4) nuclear pleomorphism (>10/10 HPF); and (5) presence of necrosis. Immunohistochemically, MAME tumors exhibit features similar to basal-like carcinomas, with expression of CK5/6, CK14, SMA, P63, S100, cadherin, GFAP, and other markers. The adenoepithelial component typically shows strong positivity for CK5/6, while the myoepithelial component may show mild or no expression. Estrogen receptor (ER) and progesterone receptor (PR) are usually negative or only focally positive, while HER2 expression is generally absent.

Current treatment consensus recommends complete excision of the tumor as the preferred approach, provided negative margins can be achieved, as most patients have a favorable prognosis (8). At Hebei Provincial People’s Hospital, four cycles of paclitaxel liposome and cyclophosphamide (TC regimen) were administered post-surgery, with no signs of recurrence or metastasis during a 9-month follow-up (9). The most secure treatment plan for MAME or recurrent AME is total excision of the affected breast, followed by close monitoring as for other malignant breast tumors. At Beijing Friendship Hospital of Capital Medical University (BFHCMU), two cycles of docetaxel, epirubicin, and cyclophosphamide (TEC regimen) were administered for recurrent MAME; however, the tumor did not show significant shrinkage (10). The role of radiotherapy and chemotherapy in the management of MAME remains unclear and warrants further investigation, with additional case reports and long-term follow-up data needed.

Given the lack of expression of ER and PR in most cases, adjuvant endocrine therapy is typically not considered. Since axillary lymph node metastasis is rare in reported cases, axillary lymph node dissection (ALND) is generally not recommended unless clinically positive lymph nodes are identified. However, two cases of pathological node positivity despite negative clinical axillary examination have been reported, suggesting that sentinel lymph node biopsy (SLNB) should be routinely performed in MAME patients. In the current case, the patient, diagnosed with low-grade malignant adenomyoepithelioma, underwent total mastectomy and SLNB, without further adjuvant treatment. The patient has remained free of recurrence and metastasis during follow-up.

Although most cases of breast AME are benign, malignant adenomyoepithelioma has the potential for distant metastasis. Most recurrent tumors are larger than 2 cm in size and tend to present with irregular margins (3–5). Ahmed and Heller (11) reviewed 11 cases of MAME and found that 4 had developed distant metastasis; Trojani et al. reported a case of lung metastasis (12), and Loose et al. documented both lung and brain metastases in one of their two cases (13). Bult et al. described a rare case of thyroid metastasis 12 years after surgery (14). Approximately 40% of MAME cases are associated with metastasis (7), with metastases typically occurring in tumors larger than 2 cm and most often within 4 months to 3 years of diagnosis. These metastases primarily spread via the bloodstream, with the lungs being the most common target organ.

## Conclusion

In summary, MAME is a rare and complex tumor with unpredictable diagnostic, treatment, and prognostic outcomes. Accurate diagnosis requires detailed histology, immunohistochemistry, and molecular testing to guide appropriate treatment. Complete local excision with SLNB is recommended as the primary treatment, given the absence of established guidelines for diagnosis and treatment, and limited clinical experience with adjuvant therapy. For cases with clear carcinomatous transformation, treatment may follow the standard protocol for breast cancer. Given the molecular and immunophenotypic similarities between MAME and triple-negative breast cancer (TNBC), exploring whether MAME could be treated using protocols established for TNBC is a subject worth further investigation.

## Data Availability

The raw data supporting the conclusions of this article will be made available by the authors, without undue reservation.
